# Lung cancer exosomes as drivers of epithelial mesenchymal transition

**DOI:** 10.18632/oncotarget.10243

**Published:** 2016-06-23

**Authors:** Mohammad A. Rahman, Jennifer F. Barger, Francesca Lovat, Min Gao, Gregory A. Otterson, Patrick Nana-Sinkam

**Affiliations:** ^1^ Division of Pulmonary, Allergy, Critical Care and Sleep Medicine, The Ohio State University, Columbus, Ohio 43210, USA; ^2^ Department of Molecular Virology, Immunology and Medical Genetics, The Ohio State University, Columbus, Ohio 43210, USA; ^3^ Liquid Crystal Institute and Chemical Physics Interdisciplinary Program, Kent State University, Kent, Ohio 44242, USA; ^4^ Division of Medical Oncology, James Comprehensive Cancer Center, The Ohio State University, Columbus, Ohio 43210, USA

**Keywords:** exosomes, vimentin, EMT, metastasis, lung cancer

## Abstract

Exosomes, a subgroup of extracellular vesicles (EVs), have been shown to serve as a conduit for the exchange of genetic information between cells. Exosomes are released from all types of cells but in abundance from cancer cells. The contents of exosomes consist of proteins and genetic material (mRNA, DNA and miRNA) from the cell of origin. In this study, we examined the effects of exosomes derived from human lung cancer serum and both highly metastatic and non-metastatic cells on recipient human bronchial epithelial cells (HBECs). We found that exosomes derived from highly metastatic lung cancer cells and human late stage lung cancer serum induced vimentin expression, and epithelial to mesenchymal transition (EMT) in HBECs. Exosomes derived from highly metastatic cancer cells as well as late stage lung cancer serum induce migration, invasion and proliferation in non-cancerous recipient cells. Our results suggest that cancer derived exosomes could be a potential mediator of EMT in the recipient cells.

## INTRODUCTION

Exosomes are ~40 to 100 nm sized vesicles [1] consisting of a lipid bilayer generated from secretory multivesicular bodies (MVB) that in turn fuse with the plasma membrane for release into the extracellular environment [2]. Exosomes are released by a wide range of cells including mast cells [3], dendritic cells [4], tumor cells [5], epithelial cells [6] and B-cells [7]. Exosomes have also been detected in a number of human body fluids, including plasma [8], urine [9], breast milk [10], amniotic fluid [11], malignant ascites [12] and bronchoalveolar lavage fluid [13], suggesting their importance in vivo. Exosomes contain various molecular constituents of their cell of origin including lipids, proteins, messenger RNA (mRNA) and micro-RNA (miRNA) all of which may be transferred from donor to target cells facilitating direct cell-to-cell contact and subsequent reprogramming of the tumor microenvironment [14]. It has been reported that exosomes derived from cancer cells can promote angiogenesis, invasion, and proliferation in recipient cells to support tumor growth and a pro-metastatic phenotype [15]. In pathological states, such as cancer, exosomes cross-talk and/or influence epithelial mesenchymal transition (EMT) [16]. EMT is the process by which epithelial cells dramatically alter their shape and motile behavior as they differentiate into mesenchymal cells [17].

Vimentin is an intermediate filament protein whose expression correlates with increased metastatic disease, reduced patient survival and poor prognosis across multiple tumor types [18]. Vimentin proteins have been implicated in many aspects of cancer initiation and progression, including tumorigenesis, EMT, and the metastatic spread of cancer [19]. The possibility that the metastatic process could be activated via exosomes in lung cancer has not yet been studied. In this study, we isolated and characterized exosomes from lung cancer cell lines as well as from human serum following a standard protocol for isolation [20]. We also examined the effect of cancer-derived exosomes on human bronchial epithelial cells (HBECs). We found that exosomes derived from highly metastatic lung cancer cells and human late stage lung cancer serum induced vimentin and EMT in recipient cells. Our findings suggest that exosomes might be a potential driver of metastatic lung progression and vimentin could be an activator of exosome-induced metastasis in human lung cancer.

## RESULTS

### PC14HM cells have higher migratory and invasive capability compared to PC14 cells

**Figure 1:**
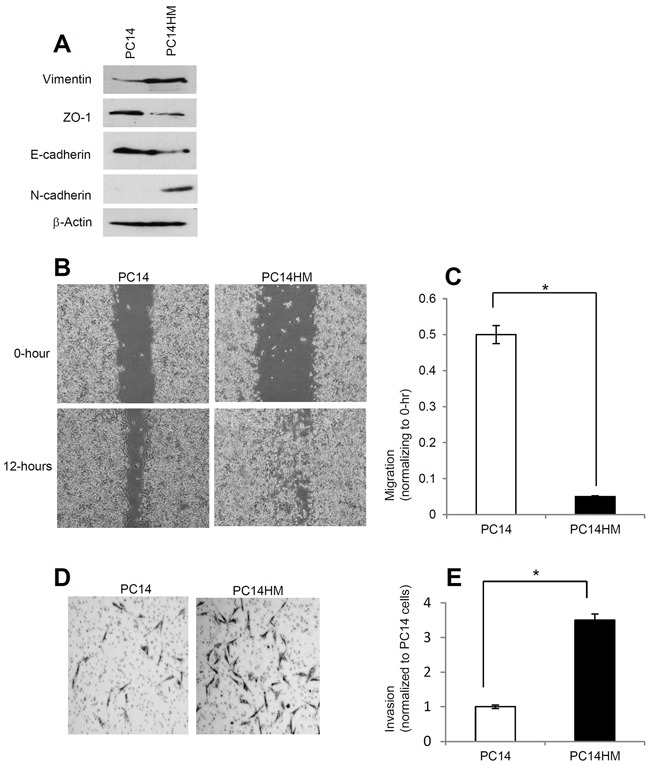
PC14HM cells show more mesenchymal properties and show more migratory as well as invasive capacity compared to PC14 cells **A.** Western Blot analysis for epithelial and mesenchymal markers in PC14 and PC14HM cell lysate samples. Twenty micrograms of total protein were analyzed by Western Blot using Vimentin, E-cadherin, ZO-1, and N-cadherin markers. β-Actin was used as an internal loading control. **B.** The *in vitro* wound healing motility assay in PC14 and PC14HM cells was performed as described in Materials and Methods. Cells were analyzed with a live cell microscope equipped with SC100 10.6 MP CMOS Color digital camera and Analysis software (Universal Imaging) (×100). **C.** Quantification of wound width between PC14 and PC14HM cells. The bars represent normalized wound width values with mean ± SD. *p<0.01 (PC14 vs PC14HM). **D.** Matrigel invasion assays were performed with the indicated PC14 and PC14HM cells. Invaded cells were stained with 0.2% crystal violet. Representative images of the bottom membrane surface are shown (40× magnification). **E.** The number of invading cells for both PC14, and PC14HM, were counted under a light microscope and statistically analyzed. *p<0.01 (PC14 vs PC14HM). Values are mean ± SD, all values are representative of at least three independent experiments.

PC14 cells are a tumorigenic but non-metastatic lung cancer cell line while PC14HM cells are highly metastatic. In PC14HM cells we found higher expression of mesenchymal markers such as vimentin, and N-cadherin compared to PC14 cells (Figure 1A). We also found lower expression of epithelial markers like ZO-1, and E-cadherin in PC14HM cells compared to PC14 cells (Figure 1A). We next examined cell migration of PC14 and PC14HM cells by wound healing assay. The highly metastatic PC14HM cells showed higher migratory capacity compared to PC14 cells (Figure 1C, 1D). Moreover, PC14HM were more aggressive and able to migrate through an extracellular matrix compared to PC14 using an invasion assay (Figure 1C, 1D). These findings suggest that PC14HM cell lines harbor more characteristics consistent with epithelial mesenchymal transition (EMT) compared to PC14 cells. Thus, we sought to characterize and examine the effect of exosomes derived from PC14 and PC14HM cells on non-malignant recipient cells.

### PC14HM cell derived exosomes express higher vimentin expression

**Figure 2:**
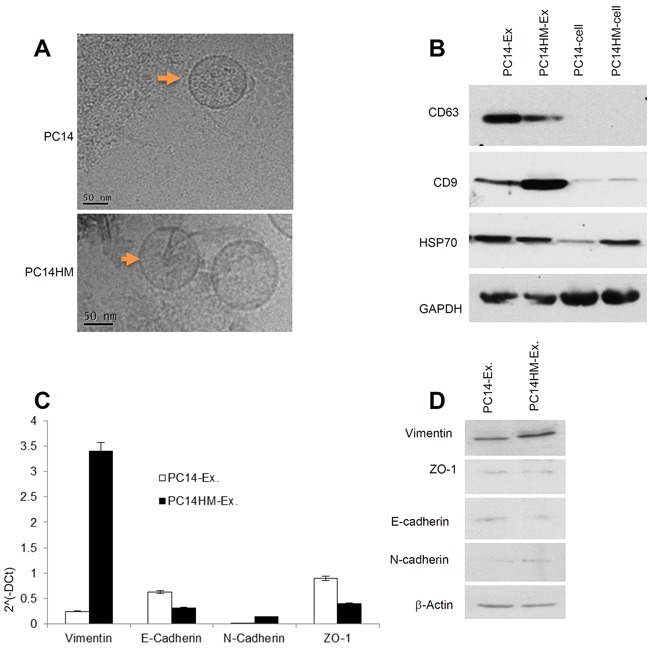
Characterization of exosomes derived from PC14 and PC14HM cells **A.** Cryo-Transmission Electron Microscopy (cryo-TEM). TEM images of exosomes derived from PC14 and PC14HM cells. **B.** Western Blot analysis for exosomes marker in exosomes and cell lysates from PC14 and PC14HM cells. Twenty micrograms of total protein from exosomes or cell lysate were analyzed by Western Blot using different exosome markers. GAPDH was used as an internal loading control. **C.** The relative mRNA expression of epithelial (E-cadherin, ZO-1), and mesenchymal (N-cadherin, Vimentin) markers by qRT-PCR in exosomal RNA isolated from PC14 and PC14HM cells. Normalization with housekeeping gene (GAPDH). The bars represent as mean ± SD of experiment performed in triplicate. **D.** Western Blot analysis for EMT marker in exosomal proteins. Twenty micrograms of total protein associated with exosomes were analyzed by Western Blot. β-Actin was used as an internal loading control. Ex indicates exosomes.

Exosomes purified from these two cell lines by serial ultra-centrifugation were identified by transmission electron microscopy to be small (30–100nm) spherical vesicles (Figure 2A). To ensure that we isolated exosomes from our preparations, we conducted Western blotting to confirm the presence of several common exosome markers, including CD63, CD9 and HSP70 (Figure 2B). We then examined exosomes for both epithelial and mesenchymal markers by qRT-PCR (Figure 2C) and Western blot (Figure 2D). Vimentin expression was significantly higher in PC14HM exosomes both at messenger and protein levels (Figure 2C, 2D).

### NanoSight tracking analysis (NTA) suggests that isolated vesicles were mostly exosomes (40~100nm)

**Figure 3:**
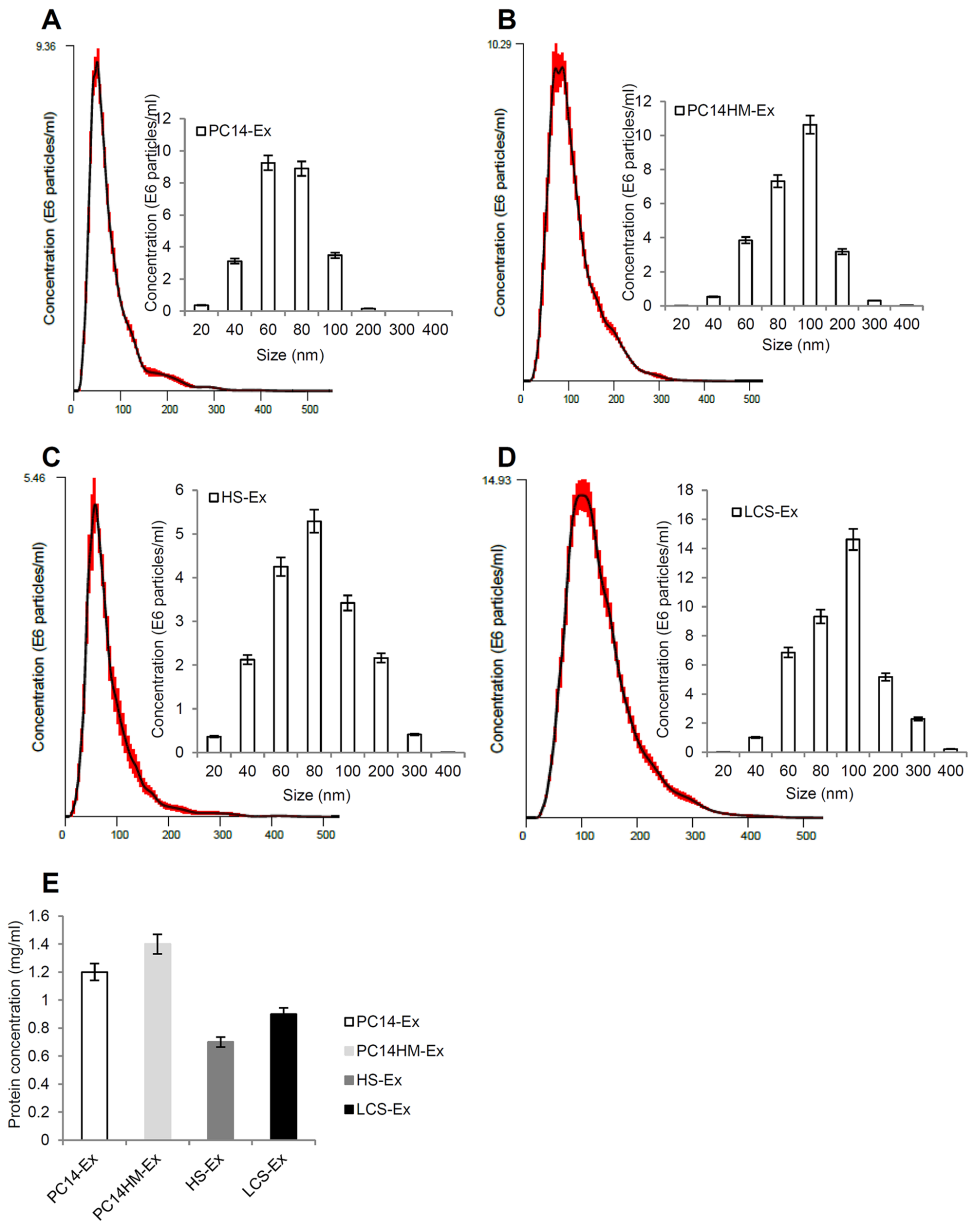
Exosome characterization by nanoparticle tracking analysis Bar chart showing the average percentage of nanoparticles within 20–300 nm size in in vitro exosome preparation. Concentration and size distribution of exosomes derived from **A.** PC14, **B.** PC14HM, **C.** healthy human serum, (HS), and **D.** lung cancer serum (LCS) were measured by nanoparticle tracking analysis (NTA). Exosomal concentration showed a peak at 60 +/− 0.5 nm (PC14 cell derived exosomes, PC14-Ex), 100 +/−0.2 nm (PC14HM cell derived exosomes, PC14HM-Ex), 80 +/−0.3 nm (healthy serum derived exosomes, HS-Ex) and 100 +/−0.7 nm (lung cancer serum derived exosomes, LCS-Ex). Bar Chart showing the particle number/ml of PC14, PC14HM, HS and LCS derived exosomes. **E.** Protein Concentration of exosomes derived from PC14, PC14HM, healthy serum (HS) and lung cancer serum (LCS). Values are mean ± SD, all values are representative of at least three independent experiments with four replicates.

NTA was used to characterize the size and estimated number/ml of isolated nanoparticles from both cell lines as well as human serum. We measured the average size distribution of nanoparticles isolated from PC14, PC14HM, human healthy serum (HS), and human lung cancer serum (LCS) using our isolation technique (Figure 3A, 3B, 3C, 3D). The curves demonstrate that the average number of nanoparticles/ml measured using the NTA system was 9.4 × 10^6^ for PC14-Ex (exosomes derived from PC14 cells), 10.3 × 10^6^ for PC14HM-Ex (exosomes derived from PC14HM cells), 5.5 × 10^6^ for HS-Ex (exosomes derived from healthy serum), and 14.9 × 10^6^ for LCS-Ex (exosomes derived from lung cancer serum) (Data were compiled from five measurements per biological replicates (n = 3). Protein concentration of exosomes was measured using a BCA assay (Figure 3E).

### PC14HM-Exosomes induce migration, invasion, proliferation and vimentin in HBECs

**Figure 4:**
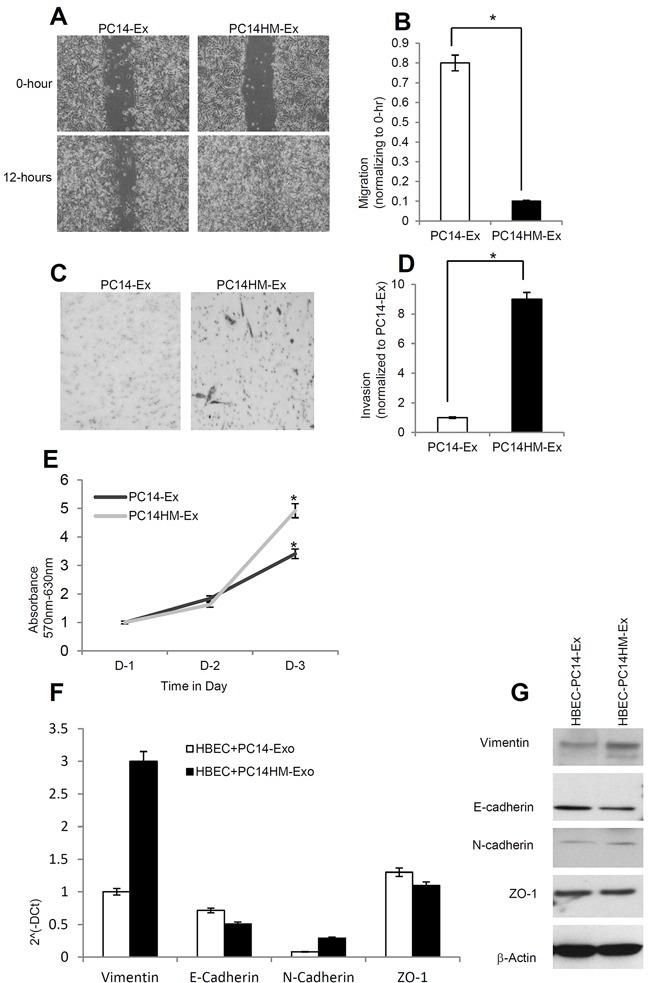
Exosomes from highly metastatic cells induce migration, invasion and vimentin expression in recipient HBEC cells **A.** Representative images from the wound healing motility assay. Confluent HBEC cells were treated with PC14 cell line derived exosomes (PC14-Ex) and PC14HM cell line derived exosomes (PC14HM-Ex) at 20 μg/μl concentration for 16 hrs. The cells were then scratched and imaged over indicated time point (0-hour and 12-hours). Each point on the assay represents three independent experiments. **B.** Quantification of wound healing motility assay. Cells were analyzed with a live cell microscope equipped with SC100 10.6 MP CMOS Color digital camera and Analysis software (Universal Imaging) (×100). The bars represent normalized wound width values with mean ± SD. *p<0.01 (PC14-Ex treatment vs PC14HM-Ex treatment). **C.** Matrigel invasion assays were performed with the HBECs cells treated with PC14-Ex and PC14HM-Ex. Invaded cells were stained with 0.2% crystal violet. Representative images of the lower membrane surface from Matrigel are shown (X400). **D.** The number of invading HBECs cells treated with either PC14-Ex or PC14HM-Ex were counted under a light microscope and statistically analyzed. *p<0.01 (PC14-Ex treatment vs PC14HM-Ex treatment) **E.** PC14HM-Ex treatment increases proliferation of HBEC cells. After 16 hrs of treatment with PC14HM-Ex or PC14-Ex, proliferation of the HBEC cells was assessed by EZ-Cytox cell viability assay kit. *p<0.01 (PC14-Ex treatment vs PC14HM-Ex treatment). **F.** The relative mRNA expression of epithelial (E-cadherin, ZO-1), and mesenchymal (N-cadherin, Vimentin) markers in HBECs cells treated with PC14-Ex and PC14HM-Ex. After 16 hrs of treatment with exosomes, total RNA was isolated from HBECs cells, 100 ng RNA was used to prepare cDNA synthesis, both epithelial and mesenchymal marker gene expression was assessed by qRT-PCR. The bars represent as mean ± SD of experiment performed in triplicate. Normalization with housekeeping gene (GAPDH). **G.** PC14HM-Ex induces vimentin protein expression in HBECs cells. HBEC cells were treated with PC14-Ex and PC14HM-Ex for 48 hrs and total proteins were isolated using RIPA buffer. Twenty micrograms of total protein associated with exosomes treated HBECs cells were analyzed by Western Blot using EMT markers. β-Actin was used as an internal loading control.

In order to investigate the effect of exosomes derived from a highly metastatic lung cancer cell line (PC14HM) on epithelial phenotype, we treated human bronchial epithelial cells (HBECs) with PC14-Ex and PC14HM-Ex for 16 hours and evaluated HBECs for migration by wound healing assay. The migration of HBECs into scratched areas of monolayers in the presence of PC14HM-derived exosomes was increased by 8-fold after 12 hours (Figure 4A, 4B). PC14HM-Ex also induced invasion of HBECs compared to PC14-Ex treatment (Figure 4C, 4D). We also found an induction of proliferation in HBECs treated with PC14HM-Ex compared to PC14-Ex (Figure 4E). We evaluated for the expression of both epithelial and mesenchymal markers in PC14-Ex and PC14HM-Ex treated HBECs. Vimentin expression was higher in PC14HM-Ex treated HBECs both at messenger and protein levels compared to PC14-Ex treated HBECs (Figure 4F, 4G).

### Late stage human lung cancer serum derived exosomes express higher vimentin and induce migration, invasion and EMT in HBECs

**Figure 5:**
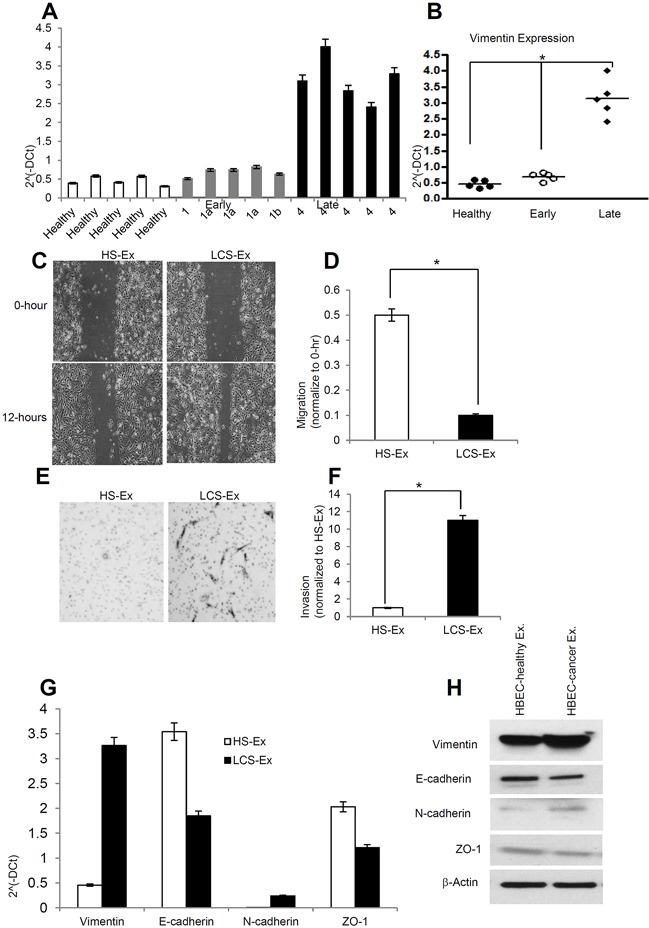
Late stage human lung cancer serum exosomes express higher vimentin and induce migration and invasion in recipient HBEC cells **A.** Vimentin expression was higher in late stage lung cancer serum exosomes compared to healthy and early stage lung cancer serum exosomes. Total 100 ng RNA was used to prepare cDNA synthesis, expression of vimentin was analyzed by qRT-PCR. Normalization with housekeeping gene (GAPDH). **B.** Quantification of vimentin expression in human lung cancer serum derived exosomes using graphpad prism software. * denotes significance higher expression of vimentin in late stage lung cancer serum derived exosomes compare to healthy and early stage lung cancer serum derived exosomes (p<0.05). **C.** Exosomes isolated from late stage lung cancer serum (LCS-Ex) induce faster migration of recipient HBEC cells as compared to exosomes isolated from healthy serum (HS-Ex). Confluent HBEC cells were treated with exosomes (at 20 μg/μl concentration) isolated from healthy serum and late stage lung cancer serum for 16 hrs. The cells then were scratched and imaged over indicated time point (0-hour and 12-hours). Each point on the assay represents three independent experiments. **D.** Quantification of wound healing motility assay. Cells were analyzed with a live cell microscope equipped with SC100 10.6 MP CMOS Color digital camera and Analysis software (Universal Imaging) (×100). The bars represent normalized wound width values with mean ± SD. *p<0.01 (HS-Ex treatment vs LCS-Ex treatment). **E.** Matrigel invasion assays were performed in HBECs cells treated with HS-Ex and late stage LCS-Ex for 24 hrs. Invaded cells were stained with 0.2% crystal violet. Representative images of the lower membrane surface from Matrigel are shown (X400). **F.** The number of invading HBECs cells treated with either HS-Ex or LCS-Ex, were counted under a light microscope and statistically analyzed. *p<0.01 (HS-Ex treatment vs late stage LCS-Ex treatment). **G.** The relative mRNA expression of epithelial (E-cadherin, ZO-1), and mesenchymal (N-cadherin, Vimentin) markers in HBECs cells treated with HS-Ex and late stage LCS-Ex. After 16 hrs of treatment with exosomes, total RNA was isolated from HBECs cells, 100 ng RNA was used to prepare cDNA synthesis, both epithelial and mesenchymal marker gene expression was assessed by qRT-PCR. The bars represent as mean ± SD of experiment performed in triplicate. Normalization with housekeeping gene (GAPDH). **H.** HBEC cells were treated with HS-Ex and late stage LCS-Ex for 48 hrs and total protein was isolated using RIPA buffer. Twenty micrograms of total protein associated with exosomes treated HBECs cells were analyzed by Western Blot using EMT markers. β-Actin was used as an internal loading control.

Exosomes were isolated from human healthy serum (n=5), early stage lung cancer serum (n=5), and late stage lung cancer serum (n=5). Twenty μg/μL exosome suspensions were used for RNA isolation. RNA was purified using an Invitrogen total RNA isolation from exosomes kit (Life science). 100 ng of RNA from healthy, early stage and late stage lung cancer serum derived exosomes was used for cDNA synthesis. Vimentin expression was normalized by GAPDH expression. Vimentin expression at the messenger level was significantly higher in exosomes derived from late stage lung cancer serum compared to healthy and early stage lung cancer serum (Figure 5A, 5B). We next sought to determine the effect of exosomes derived from human lung cancer serum on HBECs. Twenty μg/μL of exosomes derived from both healthy serum and lung cancer serum was used to test for induction of migration and invasion in recipient cells. We determined that late stage lung cancer serum derived exosomes induced migration in HBECs compared to healthy serum derived exosomes (Figure 5C, 5D). Our invasion assay also suggested that late stage lung cancer serum derived exosomes significantly increase invasion in HBECs compared to healthy patient serum derived exosomes (Figure 5E, 5F). We examined the epithelial and mesenchymal markers in HBECs treated with healthy serum exosomes as well as lung cancer serum exosomes at both messenger and protein levels. Vimentin expression was higher both at messenger and protein levels in lung cancer serum exosome treated HBECs compared to healthy serum exosome treatment (Figure 5G, 5H). N-cadherin expression was also higher in lung cancer serum derived exosome treated HBECs. Both E-cadherin and ZO-1 expression were down regulated in lung cancer serum exosome treated HBECs at the messenger and protein level. GAPDH expression was used to normalize the messenger level.

### Human lung cancer serum derived exosomes induction of EMT in bronchial epithelial cells may be mediated through vimentin

**Figure 6:**
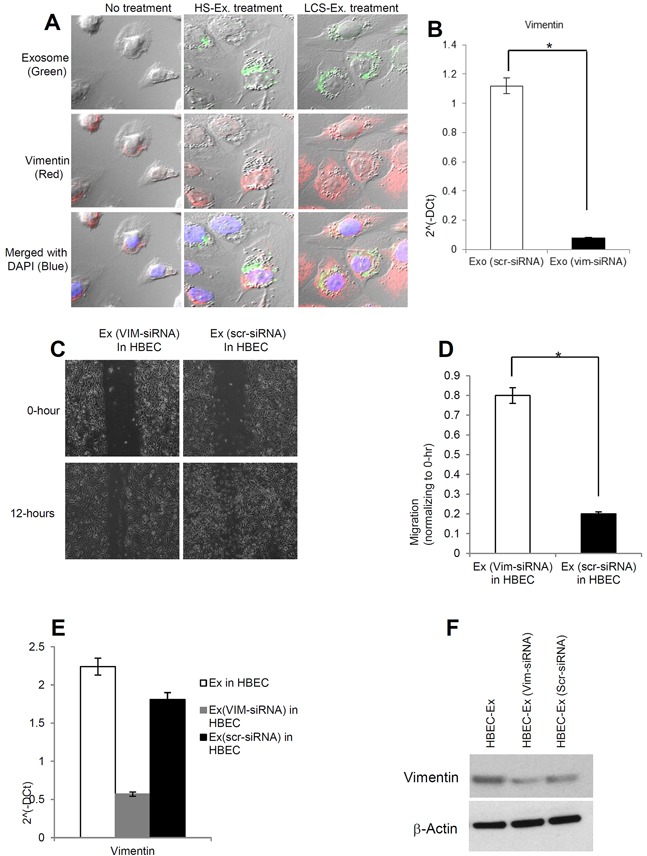
Vimentin induced by exosomes is necessary for human lung cancer to induce EMT in recipient HBEC cells **A.** PKH67-labelled (green) exosomes from healthy serum and lung cancer serum induced vimentin (red) expression in HBECs cells. HBECs cells were grown on a coverslip and treated with PKH67-labeled exosomes (20 μg/μl) for 16 hrs before fixing and staining with Vimentin antibody. HBEC cells were imaged using Confocal microscopy. Exosomes (green) internalized by HBEC cells induced vimentin (red) in HBEC cells. DAPI (blue) was used for nuclear staining. **B.** The relative mRNA expression of vimentin from exosome treated with siRNA for Vimentin (Vim-siRNA) and siRNA control (scr-siRNA). The bars represent as mean ± SD of experiment p*epr<f0o.r0m1e (dE ixno -tsrcipr-lsiciRatNe.A vs Exo-vim-siRNA). **C.** HBEC cells were grown until confluence in 6-well plate. Cells were then treated with exosomes at 20 μg/μL concentration (with and without vimentin siRNA) from human late stage lung cancer serum, and treated for 16 hrs. Representative images from three independent experiments are shown for each time point and condition. **D.** Quantification of migration assay treated with exosomes (vim-siRNA) and exosomes (scr-siRNA) in HBECs cells. Cells were analyzed with a cell live microscope equipped with SC100 10.6 MP CMOS Color digital camera and Analysis software (Universal Imaging) (×100). The bars represent as mean ± SD of experiment performed in triplicate. *p<0.01 (Ex-vim-siRNA vs Exo-scr-siRNA). **E.** The relative mRNA expression of Vimentin in HBECs cells treated with exosomes (with and without vimentin siRNA). After 16 hrs of exosomes treatment, total RNA was isolated from HBECs cells, 100 ng RNA was used to prepare cDNA synthesis, vimentin expression was assessed by qRT-PCR. The bars represent as mean ± SD of experiment performed in triplicate. Normalization with housekeeping gene (GAPDH). **F.** HBEC cells were treated with late stage LCS-exosomes, LCS-exosomes (vim-siRNA), and LCS-exosomes (scr-siRNA) for 48 hrs and total protein was isolated using RIPA buffer. Twenty micrograms of total protein associated with exosomes treated HBECs cells were analyzed by Western Blot. β-Actin was used as an internal loading control.

In order to assess for the internalization of exosomes in recipient HBECs and the effect on vimentin expression, exosomes isolated from human healthy and lung cancer serum were stained with PKH67 (green) dye and applied to HBECs. Cells were then stained for vimentin using immunofluorescence and examined under a confocal microscope. We demonstrated that vimentin expression was up-regulated in lung cancer serum derived exosome treated HBECs compared to healthy serum exosome treated HBECs (Figure 6A). In order to confirm whether the exosomal effect was mediated through vimentin or not, we targeted vimentin in exosomes through siRNA mediated knockdown and then treated HBECs. We demonstrated successful knockdown of vimentin in exosomes derived from lung cancer serum (Figure 6B). We noted a reduction in migration in HBECs when treated with vimentin depleted exosomes compared to scramble siRNA (Figure 6C, 6D) suggesting that vimentin is important for migration in HBECs. We also found that vimentin knockdown in exosomes led to decreased vimentin expression both at messenger and protein levels in HBECs (6E, 6F). These results suggest that the exosomal vimentin may contribute to cellular migration, and induction of EMT.

## DISCUSSION

The mechanisms by which extracellular vesicles may contribute to the initiation and metastatic progression of cancer remain relatively unknown. Here, we proposed that exosomes may drive EMT in lung cancer and thus, provide insight into the mechanisms of lung cancer progression and metastasis. EMT is a process that results in cancer cell migration, invasion, and metastasis with upregulation of vimentin [21, 22, 23, 24]]. Recent studies have emphasized the role of exosomes in the initiation and further metastatic progression of cancer [15]. It has been reported that a number of tumor associated macrophages (TAMs) as well as cancer associated fibroblasts (CAFs) are associated with EMT in tumor compared to normal [25]. However, the underlying molecular mechanisms are still not clear. Exosomes and their contents might play an important role in the regulation of TAMs and CAFs during EMT progression of cancer cells.

The metastatic potential of lung cancer cells is closely dependent on the tumor microenvironment [26]. To better understand the mechanisms of metastasis of human lung cancer we studied PC14HM, a human lung cancer cell line that has been determined to have high metastatic potential to multiple organs compared to the original PC14 cell line [27]. We found that PC14HM cells have a higher migratory and invasive capacity compared to the PC14 cell line. Further, PC14HM cell derived exosomes induced a more metastatic phenotype in recipient HBECs compared to PC14 cell derived exosomes. Vimentin expression was higher in PC14HM cell derived exosomes and exosome treated recipient cells compared to exosomes derived from PC14 cells. We also showed that exosomes derived from late stage lung cancer serum have higher vimentin expression and induced a metastatic phenotype in recipient cells. In order to determine if exosomal derived vimentin contributes to phenotypic changes in recipient cells, we conducted targeted siRNA mediated knockdown of vimentin. Knockdown of exosomal vimentin reduced migration in recipient cells. Exosomal vimentin knockdown also inhibited the cellular expression of vimentin compared to control knockdown. Further studies are warranted to establish the contribution of exosomes and their contents in the development of EMT in the recipient cells.

To date, very few studies in lung cancer have characterized exosomes and their role in lung cancer progression. Recent experimental studies have highlighted that exosomes can activate target cells through the transfer of ligands, receptors, and by fusion with the plasma membrane of recipient cells [28, 29, 30, 31, 32]. In addition, endocytosis of the exosomes and subsequent transfer of molecules directly into the cytosol of the recipient cell can functionally repress target genes in the recipient cells [33]. In this study, we showed that vimentin as part of exosomal contents induces EMT in recipient cells. All of these mechanisms might contribute to EMT in normal bronchial epithelial cells. Further studies are needed to establish the transformation of normal adjacent cells necessary for the metastatic process in vivo. Over the last decade, a number of studies have demonstrated that exosomes are mediators of the metastatic process. It is possible that exosomes from metastatic lung cancers contain higher vimentin (as our data suggests) and that exosomes induce vimentin in normal epithelial cells. We demonstrate that exosomes were taken up by HBECs (Figure 6A). The mechanisms that drive these observations remain unknown and will require additional investigation. To the best of our knowledge, this is the first report of lung cancer derived exosomes as well as exosomal vimentin enhancing migration in recipient cells. However, additional studies are necessary to delineate the precise mechanisms by which vimentin may contribute to cancer derived exosome-recipient cell interactions.

## MATERIALS AND METHODS

### Cell culture and transfection

The human bronchial epithelial cell (HBEC) line was obtained from ATCC (Manassas, VA) and cells were maintained in Keratinocyte-SFM medium (GIBCO, Invitrogen) supplemented with human recombinant EGF, 1% bovine pituitary extract. PC14 and PC14HM cells were kindly provided by Dr. Tetsuhiro Nakano from (Gunma University Graduate School of Medicine, Gunma, Japan) and maintained in RPMI-1640 medium (GIBCO) containing 10% FBS and 1% Penicillin/Streptomycin (Invitrogen). PC14 is a lung cancer cell line while PC14HM represent the highly metastatic cells originated from PC14 [27]. All cells were maintained at 5% CO_2_ at 37°C. All transfections were performed using siPORT according to the manufacturer's recommendations (Roche). In order to knock down vimentin, HBECs were transfected with vimentin-siRNA and scramble siRNA (Applied Biosystem) for 12 hours.

### PC14 and PC14HM cell line exosomes isolation by ultracentrifugation

PC14 and PC14HM cells were grown to70% - 80% confluence. The FBS containing media was removed, cells were washed 3X with 1X PBS and replenished with RPMI serum free medium. After 48 hours, exosomes were isolated by sequential centrifugation as described previously [34]. Briefly, the medium was collected and centrifuged at 300 × g for 10 minutes at 4°C to pellet the cells and debris followed by centrifugation at 10,000 × g for 30 minutes at 4°C. The supernatant was then ultracentrifuged at 35000 rpm for 70 minutes at 4°C. The pellet was washed with PBS and ultracentrifuged at 35000 rpm for another 70 min. The final pellet was resuspended in PBS or RIPA buffer or RNA lysis buffer. Exosome protein concentration was determined using the BCA protein assay (Fisher Scientific), and 20 μg/μl concentration of the exosome suspension was used for all experiments.

### Human serum exosomes isolation

We isolated total exosomes from frozen human serum samples by ultracentrifugation with modification. Written consent was obtained from all subjects in accordance with approved Institution Review Board protocol (1978H0059) and HIPAA regulations. Patient's information was provided in Table-1 (Supplementary Table-1). The sample was thawed quickly in a 37°C water bath and then kept on ice. Briefly, 1 ml of cell free serum was centrifuged at 2000 × g for 20 min at room temperature to remove debris, the supernatant containing the partially clarified plasma was transferred to a new tube and 5 volumes of 1X PBS was added. The serum diluted in PBS was filtered through a 0.22 μm filters (Merck Millipore) to remove contaminating apoptotic bodies, microvesicles and cell debris. The filtered-follow-through was subjected to ultracentrifugation at 55,000 rpm for 2 hrs. The pellet was wash with 1 ml PBS and ultracentrifuge at 55,000 rpm for another 2 hrs. The final pellet was resuspended in PBS or RIPA buffer or RNA lysis buffer. Exosome protein concentration was determined using the BCA protein assay (Fisher Scientific), and 20 μg/μl concentration of the exosome suspension was used for all experiments.

### Nanoparticle tracking analysis (NanoSight)

Vesicles were analyzed by nanoparticle tracking, using the NanoSight NS300 system (Malvern, Great Malvern, UK). Samples were administered and recorded under controlled flow, using the NanoSight syringe pump and script control system, and for each sample, 5 videos of 60 seconds duration were recorded, with a 10-second delay between recordings, generating 5 replicate histograms that were averaged. Therefore, the typical number of completed tracks per sample was approximately 1,200. The area under the curve was calculated using Prism-4 software version 4.03 (Graph Pad, San Diego, CA), to give average particle counts from these replicates. Particles ranging in size from 40-100 nm were considered exosomes.

### Cryo-transmission electron microscopy (Cryo-TEM)

Cryo-TEM was used as described previously [35]. To image the exosomes, 4 μL of sample (exosome in PBS) was applied to glow discharged lacey carbon coated copper grids (400 mesh, Pacific Grid-Tech, San Francisco, CA) and flash-frozen in liquid ethane within the controlled environment (22°C and 95% relative humidity) of an automated vitrification device (FEI Vitrobot Mark IV, FEI, Hillsboro, OR). The vitrified samples were transferred to a Gatan Cryo holder (Model 626.DH) and visualized in a FEI Tecnai G2 F20 ST TEM (FEI, Hillsboro, OR). The microscope was operated at 200 kV and under low dose mode to minimize radiation damage to the samples. Images were captured using a 4k × 4k Gatan Ultrascan CCD camera at a magnification of 43,000X

### Western blot analysis

Western blotting was performed on the total protein extracts from exosome samples. RIPA lyses buffer (150 mM NaCl, 50 mM Tris, pH 8.0, 5 mM EDTA, 0.5% sodium deoxycholate, 0.1% SDS, 1.0% Nonidet P-40) with protease and phophatase inhibitor cocktails (Sigma-Aldrich, St. Louis, MO, USA) was used for the total protein fraction. Protein concentrations in cell extracts as well as exosomes were determined using the BCA protein assay (Thermo-Fisher). 20 μg of total lysates were diluted 1:1 in RIPA SDS-PAGE sample buffer, loaded onto polyacrylamide gels, and blotted onto polyvinylidene difluoride membranes (Bio-Rad). Membranes were blocked with 5% non-fat milk in PBS, pH 7.6, 0.2% Tween-20 for 1 hour and then incubated with anti-CD63 (1:1000, Santa Cruz) rabbit polyclonal, anti-CD9 (1:1000, Santa Cruz) rabbit polyclonal, anti HSP70 (1:1000, Santa Cruz), anti-vimentin (1:1000, Santa Cruz) mouse monoclonal, anti-β-catenin (1:1000, Cell Signaling) mouse monoclonal, anti-ZO-1 (1:1000, Cell Signaling) mouse monoclonal, anti-E-cadherin (1:1000, Cell Signaling) mouse monoclonal, and anti-N-cadherin (1:1000, Cell Signaling) mouse monoclonal primary antibodies for overnight. After washing in TBS-T, pH 7.6, 0.2% Tween-20, the membranes were incubated with a horseradish peroxidase-conjugated goat anti-rabbit antibody (1:2000, Cell Signaling) or anti-mouse antibody (1:2000, Cell signaling), and the immunoblots were visualized using ECL detection kits, (Pierce; Rockford, IL, USA). A mouse anti-β-actin antibody (1:2000, Santa Cruz) or, anti-GAPDH (1:5000) was used as the control for equal loading of total lysate.

### Migration assay

PC14, PC14HM, and HBECs were seeded and cultured in a 6-well plate until confluence. Cells were wounded by scratching with a sterile pipette tip. Cells were then treated with exosomes (at 20 μg/μl concentration) isolated from human serum or PC14 and PC14HM cell lines or PBS. Images were captured immediately and serially at 6 and 12 hours. Migration was quantified as the average length of the elongation of wound edges over 12 hours by using Studio Lite, version 1.0 (Better Light, Inc., San Carlos, CA).

### Trans-well invasion assay

Matrigel chambers (Corning BioCoa Matrigel Invasion Chambers) were used to determine the invasiveness per the manufacturer's protocol as described previously [36]. In brief, PC14, PC14HM, and HBECs were harvested, resuspended in serum-free medium, and then transferred to the hydrated Matrigel chambers (~25,000 cells per well). The chambers were then incubated for 24 hours in culture medium with 10% FBS (for PC14 and PC14HM cells), human pancreatic serum (1%) with EGF in the bottom chambers before examination. HBECs were incubated with or without exosomes (20 μg/μl) derived from PC14, PC14HM, or human serum. The cells on the upper surface were scraped and washed away, whereas the invading cells on the lower surface were fixed and stained with 0.2% crystal violet for 10 minutes. Finally, invaded cells were counted under a microscope and the relative number was calculated.

### Proliferation assay

2×10^3^ HBECs were seeded in a 96-well plate and incubated overnight, and treated with exosomes (at 20 μg/μl concentration) or PBS. Cell proliferation was measured using the EZ-Cytox cell viability assay kit according to the manufacturer's instructions (Daeil Labservice, Seoul, Korea).

### RNA isolation

Total RNA for real-time quantitative PCR (qRT-PCR) detection analysis was extracted from cells and exosomes using TRIzol reagent according to the manufacturer's instructions (Invitrogen). RNA was cleaned with the total RNA isolation kit (Invitrogen). RNA concentration was determined by Nanodrop.

### qRT-PCR- cDNA synthesis

Synthesis of cDNA from total RNA (100 ng for mRNA) was performed using a commercially available kit (Applied Biosystem, Foster City, CA). Reverse transcription thermo cycling parameters were as follows: 25°C for 10 min, 37°C for 60 min, 37°C for 60 min 85°C for 5 min for mRNA; Reactions were performed on a MyCycler (Bio-Rad, CA, USA). Real-time PCR was performed using the ABI 7900HT system in 10 μL reaction volumes containing 1 μL of each primer, 5uL of 2× iQ™ SYBR Green Supermix, and 2 μL of nuclease-free water, 1 μL of cDNA (total 10 μL) in optical 384-well plates (Applied Biosystems). Cycling conditions: 95°C for 10 minutes, followed by 40 cycles of 95°C for 15 seconds and 60°C for 60 seconds. Triplicate qPCR reactions were performed for each cDNA sample for all experiments. The threshold fluorescence level was set manually for each plate using SDS software version 2.3 (Applied Biosystems). Following the export of Cycle threshold (Ct) data, further data analysis for both platforms was performed in Microsoft® Excel 2003. Comparison of slope and R2 values between pre-amplified and non-amplified cDNA, as a template on the BioMark arrays, was performed as a paired t-test in Microsoft® Excel 2003.

### Exosome labeling and tracking in HBECs

In order to track uptake of exosomes by human bronchial epithelial cells (HBECs), exosomes were labeled with fluorescent dye the PKH-67 labeling kit (Sigma) [37]. Briefly, 100 μg protein equivalents of exosomes were resuspended in 100 μl PBS and mixed with 100 μl of PHK67 dye diluted in diluent C (1:1 v/v) for 5 min. This mixture was diluted with 4.5 ml of PBS and centrifuged at 100,000×g for 70 min to pellet the PKH-67 labeled exosomes. The exosome pellet was further washed twice with PBS by ultracentrifugation at 100,000×g for 70 min, to remove any free dye and finally the exosome pellet was resuspended in 100 μl PBS and used for uptake studies. PKH67- stained exosomes were added to HBEC cells in culture. After 16 hours, cells were washed twice with PBS, fixed with 4% paraformaldehyde and stained with DAPI (4,6 diamidino-2-phenylindole). Exosomes were visualized using an Olympus CX60 microscope (Tokyo, Japan).

### Immunofluorescence staining of vimentin

In order to visualize vimentin, 4 × 10^4^ HBECs were seeded on sterile cover slips. PKH67 green fluorescent labeled exosomes were added to the cells and incubated for 16 hrs. Cells were washed twice with PBS, fixed with 4% paraformaldehyde in PBS for 30 minutes at room temperature; immunofluorescence was performed to detect Vimentin. Briefly, cells were blocked and incubated with anti-Vimentin (1:100, Santa Cruz). The secondary antibody utilized was anti-rabbit Alexa Fluor 594–conjugated antibody (1:2000; Invitrogen). DAPI (Invitrogen, Cat. No D21490) was used for nuclear staining. The cover slips were subsequently mounted onto slides with mounting media (Aqua poly mount, Polysciences) and analyzed via confocal fluorescence microscopy (Olympus Fluoview, FV1000 Spectral Confocal). Positive and negative controls were included on all experiments.

### Knockdown of vimentin in exosomes

The Exo-Fect Exosome Transfection Reagent was used according to the manufacturer's instruction to specifically knockdown vimentin in exosomes. Briefly, vimentin-siRNA at a final concentration of 20 pmol was mixed with Exo-Fect transfection reagent in 150 μl of siRNA buffer and exosomes (100 μg protein equivalents) derived from late stage lung cancer serum and incubated for 10 min at 37°C. The transfected exosomes were resuspended in 300 μl 1x PBS.

### Statistical analysis

All experiments were carried out in triplicate and repeated at least twice. Bars indicate standard error of the mean. The statistical significance of the results was determined using Student's t-test and Anova. The data was considered significant when p<0.05.

## SUPPLEMENTARY TABLE


