# Supplementary material for: Lung cancer exosomes as drivers of epithelial mesenchymal transition
Source: Oncotarget. 2016 Jun 23;7(34):54852–66. doi: 10.18632/oncotarget.10243 (PMC5342386; doi:10.18632/oncotarget.10243)
Supplement: Supplementary file 1 [file oncotarget-07-54852-s001.pdf]

SUPPLEMENTARY TABLE

Supplementary Table S1: Patient's information used in this study

			Lung Cancer	Lung Cancer
**Variable**		**Healthy** (n=5)	**Early stage** (n=5)	**Late stage** (n=5)
Age-yr		48.4±8.08	68.8±5.8	72.2±3.56
Sex	Male	3	2	3
	Female	2	3	2
Smoking		No	Yes	Yes

Plus-minus values are means ±SE
